# Development of an Optimized Protocol for NMR Metabolomics Studies of Human Colon Cancer Cell Lines and First Insight from Testing of the Protocol Using DNA G-Quadruplex Ligands as Novel Anti-Cancer Drugs

**DOI:** 10.3390/metabo6010004

**Published:** 2016-01-15

**Authors:** Ilaria Lauri, Francesco Savorani, Nunzia Iaccarino, Pasquale Zizza, Luigi Michele Pavone, Ettore Novellino, Søren Balling Engelsen, Antonio Randazzo

**Affiliations:** 1 Department of Pharmacy, University of Naples “Federico II”, via D. Montesano 49, 80131 Naples, Italy; ilaria.lauri@unina.it (I.L.); nunzia.iaccarino@unina.it (N.I.); ettore.novellino@unina.it (E.N.); 2 Spectroscopy & Chemometrics, Department of Food Science, Faculty of Science, University of Copenhagen, Rolighedsvej 26, 1958 Frederiksberg C, Denmark; se@food.ku.dk; 3 Department of Applied Science and Technology (DISAT), Polytechnic University of Turin—Corso Duca degli Abruzzi 24, 10129 Torino, Italy; 4 Experimental Chemotherapy Laboratory, Regina Elena National Cancer Institute, 00158 Rome, Italy; zizza@ifo.it; 5 Department of Molecular Medicine and Medical Biotechnology, University of Naples “Federico II”, via S. Pansini 5, 80131 Naples, Italy; luigimichele.pavone@unina.it

**Keywords:** cell metabolomics, colon cancer, NMR spectroscopy, Multivariate statistical analysis, G-quadruplex ligands

## Abstract

The study of cell lines by nuclear magnetic resonance (NMR) spectroscopy metabolomics represents a powerful tool to understand how the local metabolism and biochemical pathways are influenced by external or internal stimuli. In particular, the use of adherent mammalian cells is emerging in the metabolomics field in order to understand the molecular mechanism of disease progression or, for example, the cellular response to drug treatments. Hereto metabolomics investigations for this kind of cells have generally been limited to mass spectrometry studies. This study proposes an optimized protocol for the analysis of the *endo*-metabolome of human colon cancer cells (HCT116) by NMR. The protocol includes experimental conditions such as washing, quenching and extraction. In order to test the proposed protocol, it was applied to an exploratory study of cancer cells with and without treatment by anti-cancer drugs, such as DNA G-quadruplex binders and Adriamycin (a traditional anti-cancer drug). The exploratory NMR metabolomics analysis resulted in NMR assignment of all *endo*-metabolites that could be detected and provided preliminary insights about the biological behavior of the drugs tested.

## 1. Introduction

In the last decades, metabolomics studies have been performed on different biofluids (e.g., plasma, serum, urine, saliva, lymph and cerebrospinal fluid) with successful results, showing applications in many areas, such as biomarker discovery, clinical studies, nutritional studies, drug efficacy and toxicity evaluations and disease diagnosis [1,2,3,4]. However, recent developments in the use of metabolomics involve the characterization and interpretation of the cell metabolome, starting from prokaryotes (especially *Escherichia coli*) to eukaryotes cell lines (yeast or mammalian cells) [5,6]. Complementary to the classic biofluid analyses, the metabolomic profiles of cells represent a powerful tool to understand how the local metabolism and biochemical pathways are influenced by pathologies and by external or internal stimuli. In particular, the metabolome analysis of cells grown *in vitro* provides important information for the development of models of biological pathways and networks. *In vitro* cell metabolomics analysis offers several advantages: experimental variables are easier to control, higher reproducibility, less expensive and easier to interpret than analysis of animal models and human subjects [7]. The use of mammalian cells is emerging in the metabolomics field in order to understand the molecular mechanism of disease progression, the cellular response to drug treatments [8] and the cell culture monitoring [9]. In particular, the identification and characterization of cancer cell metabolomic signature may play an important role in the early diagnosis as well as in the following therapeutic response, making it possible to map the drug action into metabolic pathways [10].

Colon carcinoma is the third most commonly diagnosed cancer in the world and the second most common cause of death from cancer [11]. Surprisingly, few metabolomic studies dealing with colon carcinoma cell lines are reported in the literature [12,13,14,15,16]. The analysis of metabolic profiles of this cell line provides a comprehensive assessment of the alterations in the metabolite levels in cells and can produce important information on *in vitro* actions of drugs towards their incorporation into novel therapeutic settings.

Recently, targeting of DNA secondary structures, for example G-quadruplexes, has been considered as an appealing opportunity for drug intervention in anti-cancer therapy [17]. G-quadruplex DNA (G4-DNA) structures are four-stranded helical DNA (or RNA) structures, comprising stacks of G-tetrads, which are the outcome of planar association of four guanines in a cyclic *Hoogsteen* hydrogen-bonding arrangement. From the biological point of view, G4-DNAs are widespread in the genome and they are present in the promoters of a wide range of genes, important in cell signaling, and recognized as hallmarks of cancer: c-Myc, c-Kit and K-Ras (self-sufficiency); pRb (insensitivity); Bcl-2 (evasion of apoptosis); VEGF-A (angiogenesis); hTERT (limitless replication); and PDGFA (metastasis) [18]. The G4-DNAs are also found in telomeric regions of the chromosome [19]. Telomeric DNA consists of tandem repeats of a simple short sequence, rich in guanine residues (TTGGGA). Telomeres protect the ends of the chromosome from damage and recombination, and their shortening is implicated in cellular senescence. The elongation of telomeric DNA, operated by the enzyme telomerase, leads cancer cells towards an infinite lifetime. The inhibition of telomerase, which is over-expressed in about 85% of tumors, represents the forefront of research for new effective anti-cancer drugs. Since this enzyme requires a single stranded telomeric primer, the formation of G-quadruplex complexes by telomeric DNA inhibits the telomerase activity. In this respect, it has been found that small molecules that stabilize G-quadruplex structures are effective telomerase inhibitors and can be considered as novel drugs candidates for anti-cancer therapy [20]. Recently, it has been discovered that a number of G-quadruplex ligands are exerting interesting antitumor activity *in vitro* [21,22].

**Figure 1:**
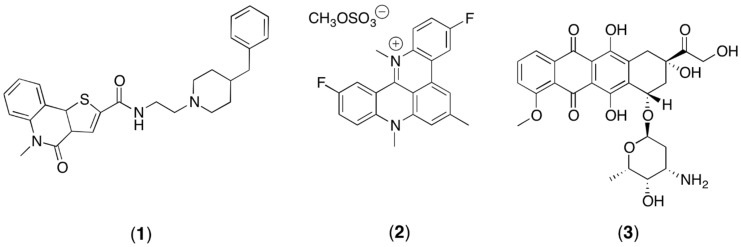
The structure of compound (**1**), RHPS4 (**2**), and the structure of the traditional antitumor agent Adriamycin (**3**).

Since the G-quadruplex ligands may be important for the development of new anti-cancer agents, this study is aimed to verify the feasibility of a NMR metabolomics study of HCT116 cells when treated with these agents. In particular, the treatment with compound **1**, which is one of the most promising ligands discovered by virtual screening calculations [23] (Figure 1), was compared to the treatment with pentacyclic acridine RHPS4 (2) (Figure 1), which is one of the most studied G4 ligands [24], and to treatment using the well-known antitumor agent Adriamycin (3) (Figure 1). Adriamycin is an approved chemotherapeutic agent with strong activity against a wide range of human malignant neoplasms including acute leukemia, non-Hodgkin lymphomas, breast cancer, Hodgkin’s disease and sarcomas [25]. Thus, this study describes an optimized protocol for NMR metabolomics of adherent mammalian cell lines and the preliminary application and validation to treated cancer cells.

## 2. Materials and Methods

### 2.1. Materials

HCT116 cells were purchased from American Type Culture Collection (ATCC–Manassas, VA, USA). High glucose Dulbecco’s Modified Eagle’s medium (DMEM/HIGH Glucose) with L-Glutamine was purchased from Euroclone (MI, Italy), penicillin–streptomycin solution for cell culture was purchased from Gibco (NY, USA). Fetal bovine serum (FBS) was purchased from Thermo Scientific (HyCloneTM). Crystal phosphate buffer saline (PBS) (0.01 M Phosphate buffer, 0.0027 M KCl e 0.14 M NaCl, pH 7.4 at 25 °C) was purchased from Bioline (TR, Italy).

Deuterium oxide (D_2_O, 99.8%D) was obtained from Sigma-Aldrich (St. Louis, MO, USA). All other reagents were of analytical grade.

### 2.2. Cell Culture

The HTC116 cells were grown in high glucose (4.5 g/L) Dulbecco’s Modified Eagle’s Medium (DMEM/HIGH Glucose, Euroclone) supplemented with 10% FBS, L-Glutamine (2 mM), penicillin (100 U/mL) and streptomycin (1 mg/mL), at 37 °C in a humidified atmosphere of 5% CO_2_. In order to obtain the final desired number of cells (1.5 × 10^6^) for each treatment, the cell growth was carried out in parallel in multiple (3×) 150 mm tissue culture dishes (Corning). Upon achievement of 90% cellular confluency, the culture medium (15 mL) was removed and the cells were processed for the endo-metabolomic analysis. In brief, the cells were extensively washed (4 times) with ice-cold phosphate-buffered saline (PBS 1X) in order to completely remove any residue of culture medium. Afterwards, 5.4 mL of PBS were added to each culture dish and cells were collected by scraping with a rubber policeman. Finally, the cells were counted, placed in Falcon tubes and the final PBS volumes were adjusted to obtain 15 × 10^6^ cells into 5.4 mL PBS (pH 7.4). On the other side, the culture medium of each cell growth was collected and immediately stored at *−*80 °C to be used, in the close future, for the exo-metabolome analyses.

### 2.3. Anti-Cancer Drug Treatments

The dose and drug exposure duration time of cell culture for compounds **1** and **2** were established according to the literature (IC_50_) [26], while the optimal conditions for compound **3** were chosen on the basis of in-house unpublished results. In order to have a unique group of untreated control cells valid for all three treatments, compounds **1** and **2** were added to cell cultures 24 h after seeding. Cells were exposed to the drug treatment for 72 h with 1 μM final concentration; compound **3** was added to cell cultures 80 h after seeding. In this case, the drug exposure of cell cultures was for 16 h with 0.1 μM final concentration. Thus, all the cells (including controls) were detached from the plates after 96 h.

### 2.4. Cell Metabolome Quenching

The Falcon tubes containing the detached cells were immersed into liquid nitrogen upon complete freezing of the samples and then slowly thawed in an ice bath. Finally, to destroy the cell membrane favoring the release of the intracellular metabolites, the quenched cells were lysed by sonication (3 short-pulse cycles of 30 s each, at maximum power).

### 2.5. Metabolites Extraction for NMR Analysis

Intracellular metabolites were extracted using a dual phase extraction procedure introduced by Bligh and Dyer in 1959 [27] with slight modifications. Adding 6 mL of cold methanol (−20 °C) and 6 mL of chloroform to the original solution (5.4 mL) containing quenched cells, briefly a mixture of water, methanol and chloroform in the volume ratio of 0.9:1:1 was obtained, corresponding to a total volume of 17.4 mL. Afterwards, this mixture containing quenched and lysed cells was incubated for 20 min on ice and vortexed frequently to facilitate the extraction. The cell extracts were centrifuged at 4000 *g* at 4 °C for 20 min. This extraction procedure generated a two-phase extract that can be described as follow: the aqueous upper phase contains water-soluble intracellular metabolites, while apolar metabolites as lipid molecules are in the organic lower phase. Proteins and macromolecules are trapped in the thin skin-like layer between the two phases. The upper and lower phase were separated and carefully transferred into different falcon tubes. Eventually, solvents were completely removed from both fraction using a vacuum concentrator (hydrophylic phase) and under a gentle flow of N_2_ gas (organic phase). Only the hydrophilic phase has been taken into account in this study while the organic phase has been stored at −80 °C for future analysis.

### 2.6. Sample Preparation for NMR Analysis

Each aqueous cell extract was dissolved in 540 μL of D_2_O together with 60 μL of a D_2_O solution containing the sodium salt of (trimethylsilyl) propanoic-2,2,3,3-d4 acid (TSP) (0.1% w/v), used as internal chemical shift reference (δ_H_ 0.00 ppm), to give a final concentration of 0.6 mM. Samples were vortexed briefly and transferred into 5-mm NMR tubes.

### 2.7. NMR Spectroscopy of Cell Extracts

All one-dimensional ^1^H-NMR spectra were acquired at 300 K on a Bruker Avance III 600 MHz ultrashielded spectrometer (Bruker Biospin Gmbh, Rheinstetten, Germany) operating at 600.13 MHz for protons (14.09 Tesla) equipped with a double tuned cryo-probe (TCI) set for 5 mm sample tubes. ^1^H NMR spectra of hydrophilic cell extracts were acquired using a one-dimensional NOESY-presat pulse sequence (RD-90°-t-90°-tm-90°-ACQ). All the experiments were acquired with an acquisition time of 2.73 s, a relaxation delay of 4 s, mixing time of 10 ms, receiver gain of 181, 128 scans, 128 K data points and a spectral width of 18,029 Hz (30.041 ppm). All samples were automatically tuned, matched and shimmed.

Representative samples of treated cell extracts were examined by two-dimensional spectroscopy (JRES, COSY, TOCSY, HSQC and HMBC) to ensure the unambiguous assignment of the metabolites. A 700 MHz Varian Unity Inova spectrometer equipped with a 5 mm ^1^H{^13^C/^15^N} triple resonance probe was used for the acquisition of two-dimensional NMR experiments.

### 2.8. NMR Data Reduction and Processing

Prior to Fourier transformation, each free induction decay (FID) was zero-filled to 128 K points and multiplied by an exponential function equivalent to a 1.0 Hz line broadening. The resulting spectra were phase and baseline corrected automatically using TOPSPIN^TM^ (Bruker Biospin) and the ppm scale was referenced according to the TSP peak at 0.00 ppm.

The NMR regions above 9.43 ppm and below 0.8 ppm were removed because they only contain noise. Furthermore, the region between 4.75 and 4.62 ppm was also removed because containing the residual water signal. Since NMR spectra showed misalignments in chemical shift due to pH-sensitive peaks, the spectra were aligned using the interval correlation optimized shifting algorithm (icoshift) [28]. A normalization preprocessing step was carried out to correct variations of the overall concentrations of the samples. Since no quantitative internal standard was used, each spectrum of the aligned NMR data matrix was normalized to unit area, obtained dividing every variable of each spectrum by the sum of the absolute value of all its variables. All preprocessing steps were performed using Matlab (2012b, The Mathworks Inc., Natick, MA, USA).

### 2.9. Multivariate Data Analysis

The normalized data matrix was imported into Simca-P 13.0 (Umetrics, Umeå, Sweden) and Pareto-scaled [29]. The number of principal components (PCs) of the Principal Component Analyses (PCA) [30] was determined by leave one out cross-validation [31]. The quality of the models was described by the squared Pearson correlation coefficient *R*^2^ and Q^2^ values. *R*^2^ is defined as the proportion of variance in the data explained by the models and indicates the goodness of fit. Q^2^ is defined as the proportion of variance in the data predictable by the model and indicates predictability [29]. Both *R*^2^ and Q^2^ vary between 0 and 1: a good prediction model is indicated by Q^2^ > 0.5, whereas a Q^2^ > 0.9 means an excellent predictive ability of the model. In this study, all PCA models performed showed a *R*^2^ ≥ 0.9 and a Q^2^ ≥ 0.8, which means goodness of fit and goodness of prediction of the models.

### 2.10. Metabolite Identification

Identification of hydrophilic metabolites was achieved by (i) comparison with the chemical shifts of the metabolites in the Human Metabolome Database (HMDB) [32]; (ii) peak fitting routine within the spectral database in Chenomx NMR Suite 5.0 software package (Chenomx, AB, Canada); (iii) analysis of literature data [33,34,35]; (iv) the interpretation of the bi-dimensional NMR spectra; and (v) the analysis of the Statistical Total Correlation Spectroscopy (STOCSY) [36].

### 2.11. Statistical Total Correlation Spectroscopy Analysis

Statistical Total Correlation Spectroscopy (STOCSY) analysis (Figure S1) was performed on the binned (0.02 ppm) NMR (1D-NOESY) data set containing all samples, to obtain the correlations among the metabolite signals. The results were plotted using a threshold value of *R* > 0.95.

### 2.12. Metabolic Pathways Identification

The impact of drug treatment of HCT116 colorectal carcinoma cell line on metabolic pathways was evaluated using a tool for metabolomic data analysis, which is available online [37]. The Pathway Analysis module combines results from powerful pathway enrichment analysis with the pathway topology analysis to help researchers identify the most relevant pathways involved in the conditions under study. By uploading the discriminatory compounds that were significantly influenced by drug treatment, the built-in *Homo sapiens* (human) pathway library for pathway analysis and hypergeometric test for over-representation analysis were employed. Results were then presented graphically as well as in a detailed table (Figure S2).

### 2.13. Statistics

Values are presented as the mean ± SD. Differences between data sets were analyzed by a one-way ANOVA, and *p* < 0.05 was considered to be statistically significant.

## 3. Results and Discussion

### 3.1. Optimization of the Quenching and Extraction Procedures

This investigation was aimed to verify the feasibility of the study of the metabolome of human colon cancer cell line (HCT116) by NMR when treated with anti-cancer drugs. Mammalian cell metabolomics is an emerging research field, however the number of studies concerning quenching and extraction methods for HCT116 cells is still limited and generally referred to studies performed by GC-MS and LC-MS.

**Figure 2:**
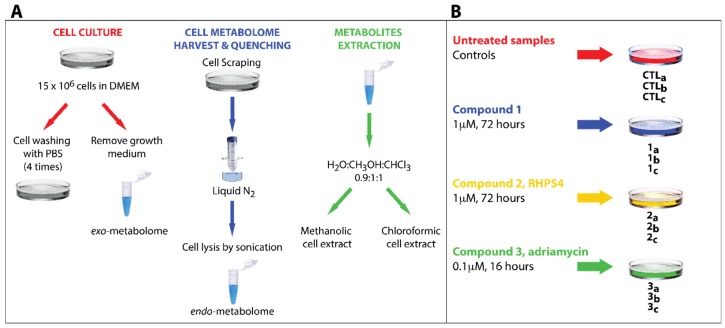
(**A**) General scheme describing the whole sample preparation protocol. (**B**) Overview of the experimental design. Each compound has been tested in triplicate and three control samples (untreated ones) were also collected (CTL_a-c_).

In this study, several published protocols for NMR-based metabolomic analysis to recover the cell metabolome were tested and the best results were achieved by selecting and combining different steps described in the diverse procedures [38] (Figure 2A). By analyzing and investigating the different extraction protocols, a number of critical passages that required an extensive optimization were identified. For example, the effects of cell quenching in liquid nitrogen were thoroughly investigated. This commonly represents the first step in several extraction protocols (immediately after the growth medium removal) just before cell washing to remove the medium residues. However, in this study it was observed that washing the HCT116 cells after the quenching step turned in to a significant loss of the cell metabolites, presumably because the freezing step induces the cell wall breakage with consequent metabolite leakage. To overcome this problem, the order of quenching and washing was inverted. Moreover, as the HCT116 cells grow as a sub-confluent monolayer, it was found particularly difficult to completely remove the cell growth medium during the washing step. In particular, one of the most abundant components of the medium, glucose, challenged the spectral interpretation of the extracted metabolome due to its residual signals spread all over the central region of the NMR spectrum. In order to avoid this, the number of washing steps was increased to four. After this intense washing procedure, the cells could be detached from the dishes by mechanical scraping and the metabolic activity of the cells immediately quenched by liquid nitrogen. The optimized protocol is summarized in Figure 2A, and can be recapitulated in the following main steps: (i) growth of the cell culture; (ii) abundant washing; (iii) cell scraping; (iv) quenching in liquid nitrogen; (v) cell lysis by sonication; and (vi) dual phase extraction procedure of the metabolites. Experimental description of these steps is reported in Material and Methods Section.

### 3.2. Experimental Design

In order to reduce bias in the interpretation of the experiments, it was decided to produce three biological replicates for each treatment (namely with compounds **1**–**3**). Furthermore, three control samples (untreated ones) were also collected (CTL_a-c_). Thus, a total of 12 samples were produced and studied by high-resolution ^1^H NMR. The whole design of experiment is summarized in Figure 2B. The most efficient dose and drug exposure duration time of cell culture were used for each compound.

**Table 1:** NMR assignment of the identified metabolites. The values indicate the percentage of increment or decrement in signal intensity of any given metabolite upon treatment with respect of the control. The values reported in talics are not statistically significant to be taken into account since the percentage of variation is less than three times the standard deviation (arbitrary threshold).

Identification Number	Metabolites	Chemical Shifts (ppm)	Compound 1	Compound 2	Compound 3
1	Lactate	1.33(d)	+19% ± 4%	+165% ± 18%	*+18 ± 10%*
		4.13(q)			
2	Threonine	1.34(d)	+14% ± 4%	−46% ± 2%	*+17 ± 8%*
		4.27(m)			
3	Tyrosine	6.91(m)	+28% ± 3%	−36% ± 3%	+17±5%
		7.21(m)			
4	Phenylalanine	7.34(d)	+23% ± 1%	−34% ± 2%	+13% ± 3%
		7.39(m)			
		7.44(m)			
5	Creatine	3.04(s)	+23% ± 2%	+49% ± 10%	+19% ± 5%
		3.95(s)			
6	Creatine phosphate	3.05(s)	*−13% ± 5%*	−55% ± 2%	*0 ± 9%*
		3.96(s)			
7	Glycine	3.58(s)	*−8% ± 4%*	−43% ± 4%	*+6 ± 9%*
8	Alanine	1.49(d)	*+2% ± 3%*	−29 ± 5%	*+15 ± 9%*
		3.81(q)			
9	Acetate	1.92(s)	*0 ± 20%*	+14% ± 1%	*0% ± 50%*
10	Succinate	2.39(s)	+7% ± 1%	+122% ± 122%	+13% ± 1%
11	AMP	4.02(dd)	*+5 ± 4%*	−36 ± 5%	*+16% ± 8%*
		4.36(dd)			
		4.51(dd)			
		8.28(s)			
		8.59(s)			
12	Isoleucine, Leucine, Valine	0.94(t)	+29% ± 4%	*−11% ± 5%*	*+15% ± 9%*
		1.02(d)			
		0.97(d)			
		0.99(d)			
		1.05(d)			
13	O-Phosphocholine	3.23(s)	−68% ± 1%	−61% ± 1%	+21% ± 7%
		4.17(m)			
14	Glycerophosphocholine	3.24(s)	−20% ± 2%	−33% ± 4%	*−1% ± 6%*
15	Nicotinic acid adenine dinucleotide (NAAD)	8.06(t)	−12% ± 2%	−48% ± 3%	+15% ± 2%
		8.15(s)			
		8.42(s)			
		8.75(d)			
		8.95(d)			
		9.13(s)			
16	NAD^+^/NADP^+^	6.10(d)	−8% ± 2%	+62% ± 11%	*+6% ± 6%*
		8.18(m)			
		8.84(d)			
		9.12(d)			
		9.32(s)			
17	Histidine	7.10(d)	+24% ± 1%	−52% ± 3%	+14% ± 2%
		7.86(d)			
18	Glutathione	2.97(dd)	+9% ± 2%	−43% ± 9%	+13% ± 4%
		4.57(q)			
		2.58(m)			
19	ATP	8.52(s)	−24% ± 7%	*−8% ± 4%*	−26% ± 6%

The limited number of independent samples (12) used in this study is not sufficient to draw general conclusions, However, this represents a significant and necessary feasibility study before setting up a much larger project. Indeed, the whole procedure described in Section 2.2, Section 2.3, Section 2.4 and Section 2.5, from cell seeding to metabolites extraction, represents by far the most labor, cost and time intensive part of the whole study. In order to achieve a satisfactory and reliable result in terms of reproducibility and growth yield many trials were conducted to perfection the presented protocol. The purpose was to demonstrate the feasibility of the NMR metabolomics approach by developing a reliable protocol for cancer cell line metabolomics using a limited number of reliable sample results.

### 3.3. Metabolic Profile

**Figure 3:**
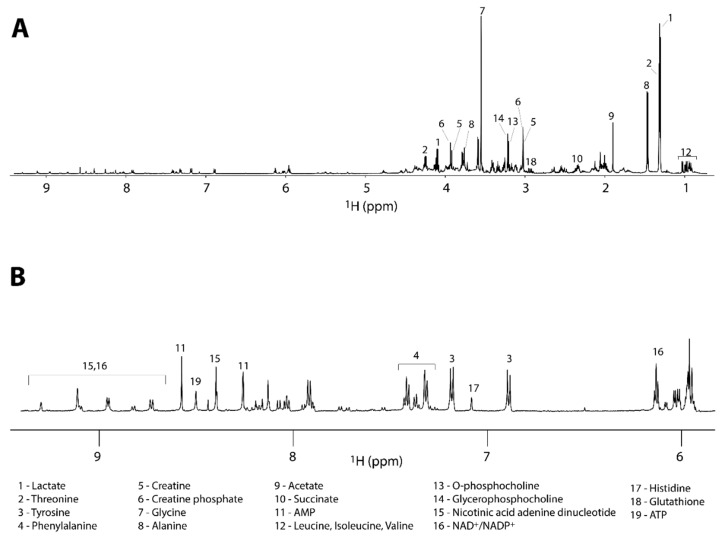
(**A**) Full ^1^H-NMR spectrum of a representative control sample along with the assignment of the most intense signals. (**B**) Expanded region of the spectrum reported in (**A**) with the assignment of the less intense metabolites.

The 1D ^1^H NMR spectra were acquired to determine the metabolic fingerprints of the treated and untreated cancer cells, while 2D homo- and hetero-nuclear NMR experiments were acquired for the assignment of the metabolites. The metabolite assignment was accomplished by comparing data from literature, by peak fitting routine within the spectral database in Chenomx NMR software package, by the analysis of available chemical shifts databases (*i.e.*, HMDB) and by STOCSY correlation analysis (Figure S1). The results of the assignment are reported in the Table 1 and in Figure 3.

**Figure 4:**
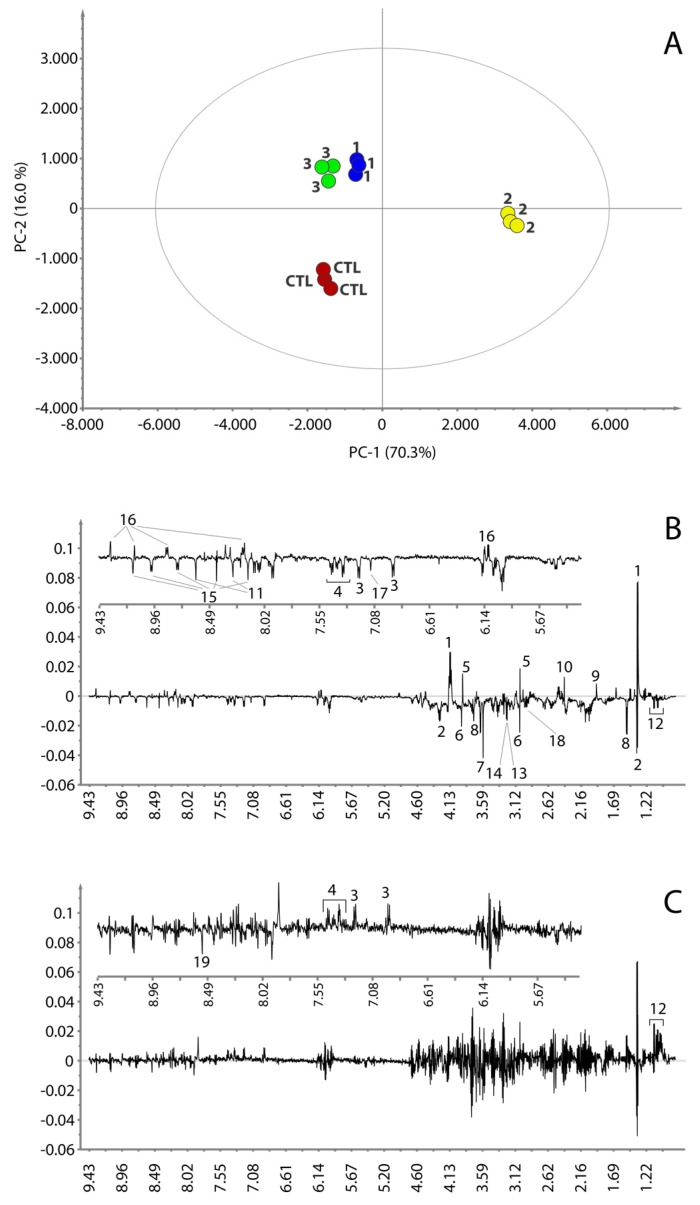
PCA score plot (**A**). PC-1 and PC-2 loading plots are reported in panel (**B**,**C**), respectively. Insets in (**B**,**C**) are expanded regions of the relative loading plots. Control samples are colored in red; compound **1**, **2** and **3** in blue, dark yellow and green, respectively. Numbers on the loading plots refer to the NMR assignment reported in Figure 3.

The 1D ^1^H NMR spectra were processed and studied using a completely untargeted and unbiased multivariate data analytical approach. The aim was to identify the commonalities in the metabolic signatures associated with response to treatment for each tested compound. For this reason, a principal component analysis (PCA) was performed on the NMR spectra. The PCA scores plot displaying the two main principal components (PCs) accounting for 86.3% of the variance (PC-1 70.3%, PC-2 16.0%) is shown in Figure 4A. The PCA scores plot shows that the samples of the cells treated with RHPS4 (**2**) are positioned on the extreme right side of the principal direction of variance PC-1 and the samples of the cells treated with **1**, **3** and controls are placed to the left. Along with PC-2, the treatments with **1** and **3**, positioned in the up-left quadrant of the plot, differ from the control samples, which are found in the bottom-left quadrant.

**Figure 5:**
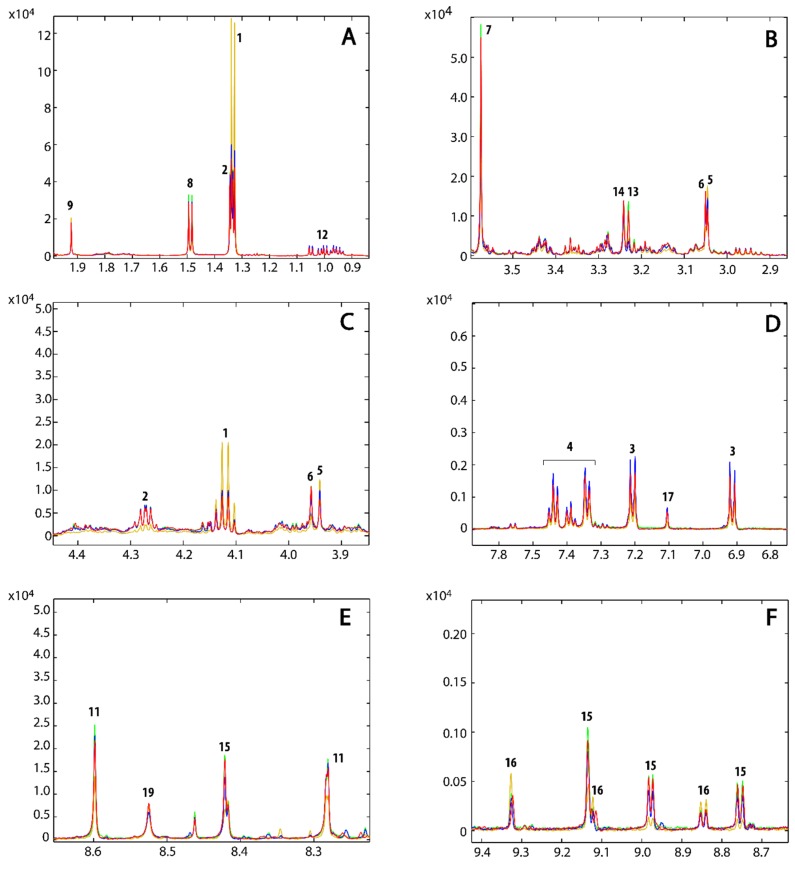
Expended regions of the superimposition of the mean NMR spectra of the untreated samples (**red**), and samples treated with compound **1** (**blue**), **2** (**dark yellow**) and **3** (**green**).

The loadings plot for the first principal component (Figure 4B) shows that the samples treated with **2** are characterized by a higher content of lactate, creatine, acetate, succinate and NAD^+^/NADP^+^, whereas the concentrations of threonine, glycine, alanine, tyrosine, phenylalanine, leucine, isoleucine, valine, histidine, creatine phosphate, glycerophosphocholine, O-phosphocholine, glutathione, NAAD and AMP are lower with the respect of the samples that lie on the left of the plot. The loadings plot of the second principal component (Figure 4C) is much noisier than that observed for PC-1. However, it appears that the samples treated with **1** and **3** differ from the control samples by having a higher content of leucine, isoleucine, valine, tyrosine, phenylalanine and a lower content of ATP. However, in order to better understand the effect of **1** and **3** on the metabolism of the cancer cells and to confirm the effect of **2**, a direct comparison of the average ^1^H NMR spectra of the three replicates for each treatment and controls was performed. The most interesting regions are reported in Figure 5. This comparison further corroborated the observation done for **2** and revealed that the treatments with **1** and **3** also caused variation in the content of lactate, threonine, glycine, creatine phosphate, glycerophosphocholine, O-phosphocholine, histidine, NAD^+^/NADP^+^ and its precursor NAAD (Figure 5). Specifically, the concentration of lactate, threonine and creatine increases both in treatments with **1** and **3**. Glycine and creatine phosphate both decrease by treatment with **1**, whereas the treatment with **3** shows only a slight increment of creatine phosphate. O-phosphocholine and glycerophosphocholine were observed to decrease upon treatment with **1**, while samples treated with **3** showed only a slight increment of O-phosphocholine. The behavior of NAAD closely resembles that of creatine phosphate and glycerophosphocholine for all three treatments. On the other hand, histidine increased in the cell extracts by treatment with compounds **1** and **3**. On the contrary, the concentration of NAD^+^/NADP^+^ increased when the cell were treated with compound **3** and decreased by treatment with **1**. Furthermore, the content of acetate and succinate does not vary, while, concentration of ATP decreases in all three treatments. The behavior of all the cell metabolites is summarized in Table 1.

### 3.4. Metabolic Pathways Analysis

As mentioned in the previous paragraph, many metabolites were affected by the treatment with compounds **1**, **2** and **3** (Table 1). In order to identify which metabolic pathways are involved, the MetaboAnalyst [37] web server was used. This tool suggests the most relevant pathways by uploading the discriminatory compounds that were significantly influenced by drug treatment. Results are provided in a so called “metabolic pathway analysis” and a “metabolite set enrichment overview” (Figure S2).

In particular, compound **2** significantly perturbs the levels of the metabolites that are involved in mitochondrial activities, compared to the untreated control cells. In fact, among the detected metabolites, the increased levels of succinate indicate inhibition of Complex I of respiratory chain of mitochondria useful to convert succinate in fumarate. This event thus impairs TCA cycle and production of ATP. Furthermore mitochondrion dysfunctions are shown by impaired conversion of creatine to creatine phosphate that results in further impairment of urea cycle and amino acid synthesis. Finally, decreased level of ATP and increased level of lactate and acetate are clear signs of apoptosis [39] and cell death in accordance with the down-regulated glutathione biosynthesis that suggest an increased reactive oxygen species (ROS) generation and a weakened ability to balance ROS. The cell death process was further supported by the reduction of choline metabolism that inhibits protein and DNA synthesis. Compound **1** behaves similarly to compound **2** because of the increase of lactate, creatine and decrease of creatine phosphate, ATP and glycine, as well as decrement of choline metabolism (glycerophosphocholine and O-phosphocholine). However, compounds **1** and **3** do not seem to interfere with TCA cycle, since succinate did not change. Compound **3**, similarly to **1** and **2**, drives cell death and apoptosis because of the increased lactate and creatine and decreased ATP.

In summary, the three tested compounds significantly altered the metabolism of the cells. The NMR data demonstrate that the treatments generally affect amino acid turnover or protein biosynthesis (alanine, glycine, isoleucine, leucine, valine, tyrosine, phenylalanine, threonine, histidine), tricarboxylic acid (TCA) cycle and mitochondrial activity (succinate, NAAD, NAD, ATP), urea cycle (creatine, creatine phosphate), anaerobic metabolism (lactate) and protein and DNA biosynthesis and DNA repair (choline and phosphocholine). Furthermore, the specific alterations in the choline metabolism by compounds **1** and **2** indicate that cell death in HCT116 lines is induced interfering with DNA synthesis and DNA damaged repair and by inhibition of protein synthesis. The NMR data thus strongly suggest that treatment with compounds **1** and **2** slow down cellular metabolism, aggravate oxidative stress and reduces DNA synthesis and repair leading to cellular death and apoptosis in accordance with their anti-cancer activity. Compound **3** also drives cell death and apoptosis due to a general cytotoxicity in accordance with anti-cancer activity of Adriamycin [40].

## 4. Conclusions

The implementation of a reliable NMR metabolomics analytical protocol has been quite challenging, owing to the number of critical steps along the way from cell culture to NMR tubes. This investigation was aimed at establishing a reliable protocol that describes how to handle the metabolome of the HCT116 human colon cancer cell line in order to perform a trustworthy metabolomic NMR analysis. This was pursued by simulating potential drug treatments using a limited number of “reliable” samples.

The best protocol was selected by combining different analytical procedures reported in the literature.

The optimized protocol can be summarized in the following main steps: (i) growth of the cell culture; (ii) abundant washing; (iii) cell scraping; (iv) quenching in liquid nitrogen; (v) cell lysis by sonication; and (vi) dual phase extraction procedure of the metabolites. It was demonstrated that the yield of the extraction and the quality of the extracted metabolome is of sufficiently high quality that the NMR assignment of detectable [41] metabolites could also be accomplished. Furthermore, preliminary insight into the biological behavior of the three tested anti-cancer compounds were accomplished.

## Supplementary Materials

The following are available online at www.mdpi.com/2218-1989/6/1/4/s1, Figure S1: (A) Full STOCSY plot colored according to the correlation coefficient (see colored bar on the right); (B–D) Expanded regions of the STOCSY reporting the main correlations for NAD/NADP, NAAD and Lactate, respectively, Figure S2: Impact of the treatment with compounds 1-3 (panel A-C, respectively) on metabolic pathways of HCT116 cell, Table S1: CHEBI codes for the identified metabolites.
