# Supplementary material for: Development of an Optimized Protocol for NMR Metabolomics Studies of Human Colon Cancer Cell Lines and First Insight from Testing of the Protocol Using DNA G-Quadruplex Ligands as Novel Anti-Cancer Drugs
Source: Metabolites. 2016 Jan 15;6(1):4. doi: 10.3390/metabo6010004 (PMC4812333; doi:10.3390/metabo6010004)
Supplement: Supplementary file 1 [file metabolites-06-00004-s001.pdf]

# Supplementary Material: Development of an Optimized Protocol for NMR Metabolomics Studies of Human Colon Cancer Cell Lines and First Insight from Testing of the Protocol Using DNA G-Quadruplex Ligands as Novel Anti-Cancer Drugs

**Ilaria Lauri, Francesco Savorani, Nunzia Iaccarino, Pasquale Zizza, Luigi Michele Pavone, Ettore Novellino, Søren Balling Engelsen and Antonio Randazzo**

**Probing the *Endo*-Metabolome of Human Colon Cancer Cell Lines by NMR Spectroscopy: Testing the Stimuli of DNA G-Quadruplex Ligands as Novel Anti-Cancer Drugs**

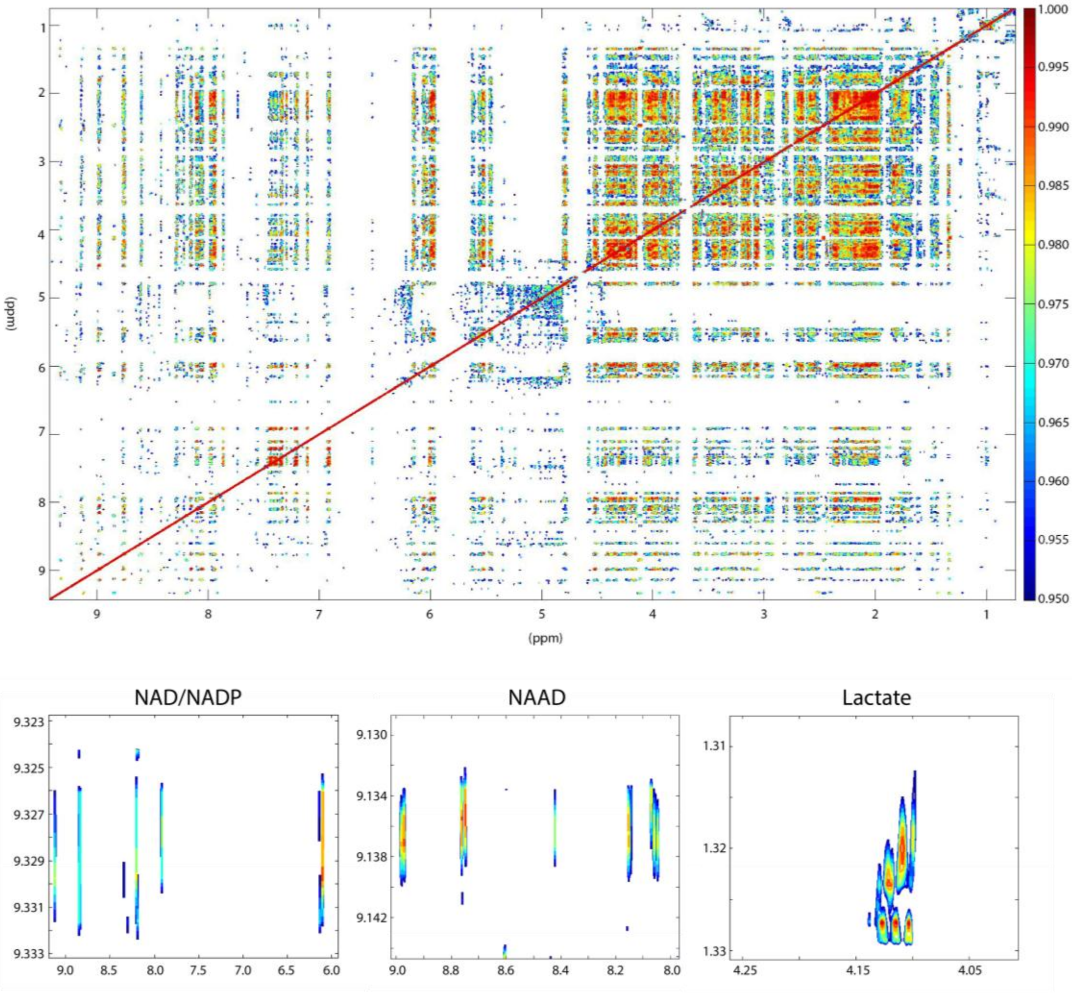


**Figure S1.** (**A**) Full STOCSY plot colored according to the correlation coefficient (see colored bar on the right); (**B**–**D**) Expanded regions of the STOCSY reporting the main correlations for NAD/NADP, NAAD and Lactate, respectively.

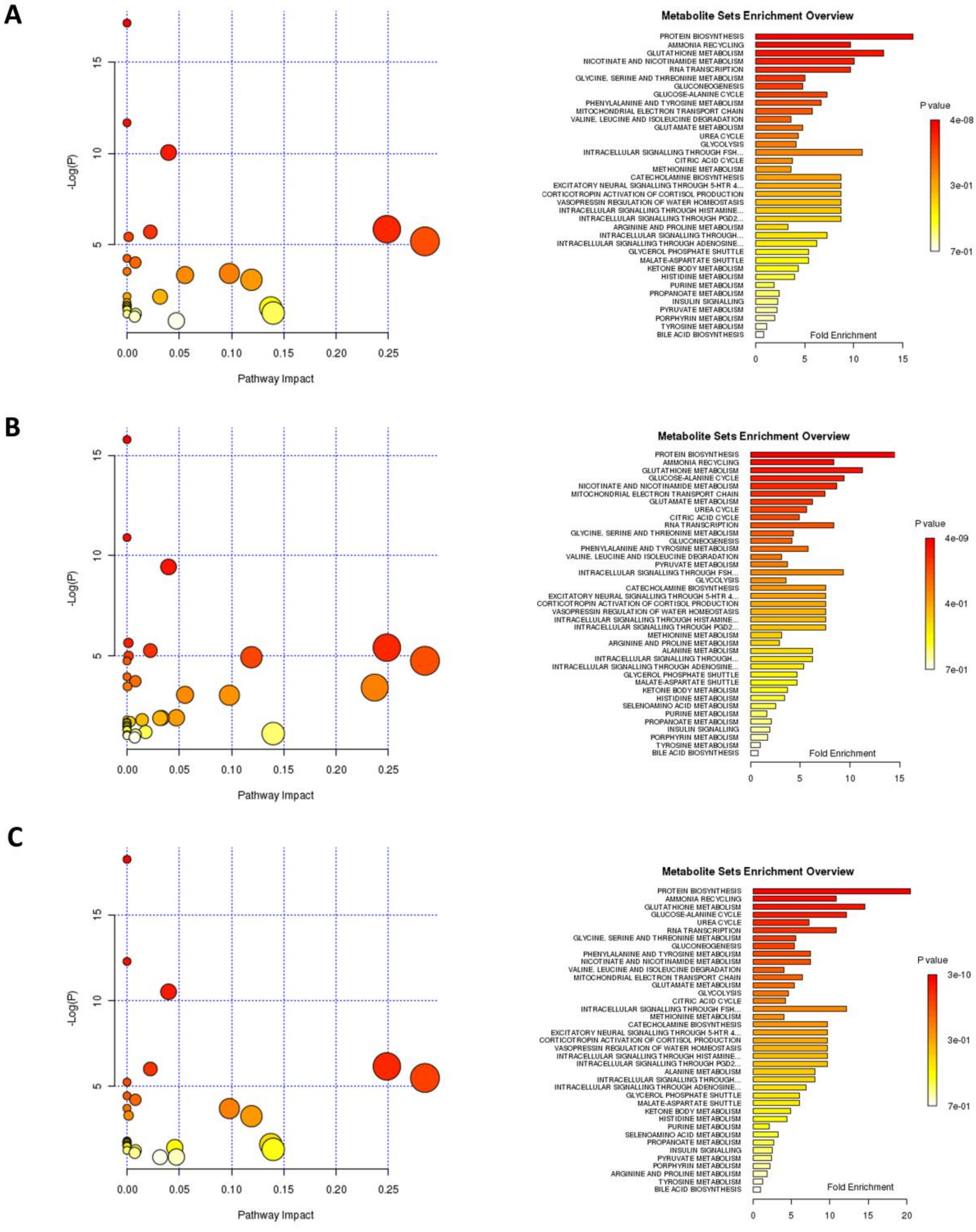


**Figure S2.** Impact of the treatment with compounds **1–3** (panel **A–C**, respectively) on metabolic pathways of HCT116 cell. Intracellular metabolite-based metabolic pathway analysis and metabolite sets enrichment overview are reported on the left and on the right, respectively.

**Table S1.** CHEBI codes for the identified metabolites.

| Identification Number | Metabolite | CHEBI Code |
|---|---|---|
| **1** | Lactate | CHEBI:16651 |
| **2** | Threonine | CHEBI:16857 |
| **3** | Tyrosine | CHEBI:17895 |
| **4** | Phenylalanine | CHEBI:17295 |
| **5** | Creatine | CHEBI:16919 |
| **6** | Creatine phosphate | CHEBI:17287 |
| **7** | Glycine | CHEBI:15428 |
| **8** | Alanine | CHEBI:16977 |
| **9** | Acetate | CHEBI:30089 |
| **10** | Succinate | CHEBI:30031 |
| **11** | AMP | CHEBI:456215 |
| **12** | Isoleucine, Leucine, Valine | CHEBI:17191<br>CHEBI:15603<br>CHEBI:16414 |
| **13** | O-Phosphocholine | CHEBI:18132 |
| **14** | Glycerophosphocholine | CHEBI:36313 |
| **15** | Nicotinic acid adenine dinucleotide (NAAD) | CHEBI:75967 |
| **16** | $NAD^+$/$NADP^+$ | CHEBI:57540<br>CHEBI:58349 |
| **17** | Histidine | CHEBI:15971 |
| **18** | Glutathione | CHEBI:16856 |
| **19** | ATP | CHEBI:30616 |
